# Selecting single cell clustering parameter values using subsampling-based robustness metrics

**DOI:** 10.1186/s12859-021-03957-4

**Published:** 2021-02-01

**Authors:** Ryan B. Patterson-Cross, Ariel J. Levine, Vilas Menon

**Affiliations:** 1 Spinal Circuits and Plasticity Unit, National Institute of Neurological Disorders and Stroke, National Institutes of Health, Bethesda, MD USA; 2 Department of Neurology, Center for Translational and Computational Neuroimmunology, Columbia University, New York City, NY USA

**Keywords:** Single cell RNAseq, Parameter selection, Clustering, Resolution

## Abstract

**Background:**

Generating and analysing single-cell data has become a widespread approach to examine tissue heterogeneity, and numerous algorithms exist for clustering these datasets to identify putative cell types with shared transcriptomic signatures. However, many of these clustering workflows rely on user-tuned parameter values, tailored to each dataset, to identify a set of biologically relevant clusters. Whereas users often develop their own intuition as to the optimal range of parameters for clustering on each data set, the lack of systematic approaches to identify this range can be daunting to new users of any given workflow. In addition, an optimal parameter set does not guarantee that all clusters are equally well-resolved, given the heterogeneity in transcriptomic signatures in most biological systems.

**Results:**

Here, we illustrate a subsampling-based approach (chooseR) that simultaneously guides parameter selection and characterizes cluster robustness. Through bootstrapped iterative clustering across a range of parameters, chooseR was used to select parameter values for two distinct clustering workflows (Seurat and scVI). In each case, chooseR identified parameters that produced biologically relevant clusters from both well-characterized (human PBMC) and complex (mouse spinal cord) datasets. Moreover, it provided a simple “robustness score” for each of these clusters, facilitating the assessment of cluster quality.

**Conclusion:**

chooseR is a simple, conceptually understandable tool that can be used flexibly across clustering algorithms, workflows, and datasets to guide clustering parameter selection and characterize cluster robustness.

## Background

**Fig. 1:**
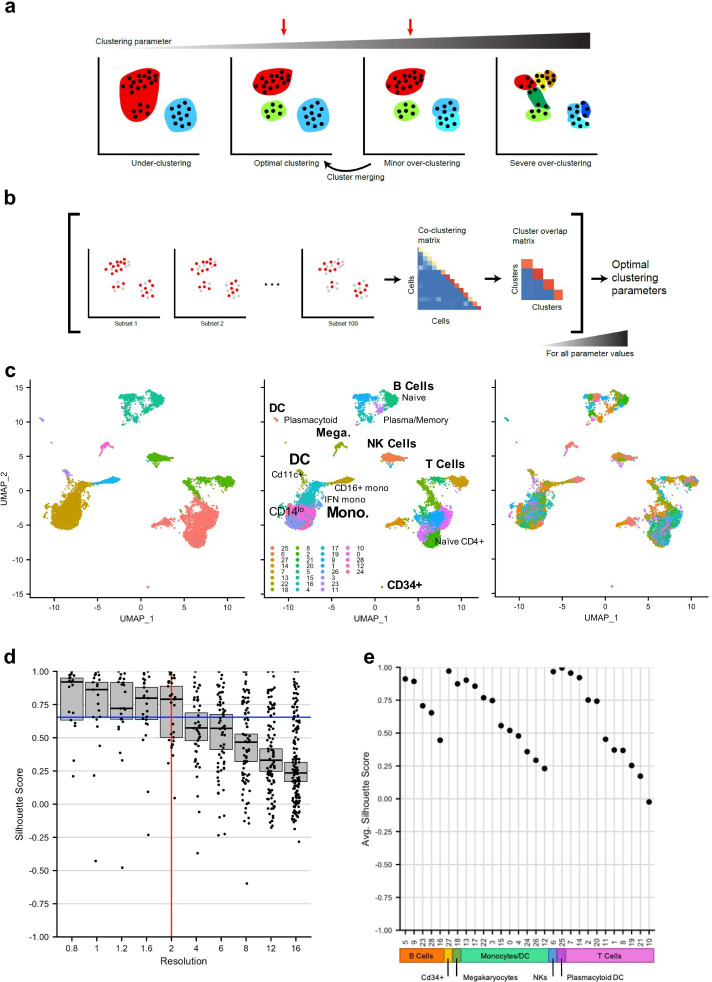
Subsampling-based robustness metrics identify near-optimal clustering parameter values. **a** Schematic of the clustering parameter selection problem. In the under-clustering case, clearly distinct biological differences remain unresolved. In the optimal clustering case, cells are appropriately grouped, while in the minor over-clustering case, some groups of cells are over-split. Either the optimal clustering or the minor over-clustering parameter regime is appropriate (red arrows), since the latter can be modified through cluster re-merging to obtain the optimal clustering. With severe over-clustering (rightmost panel), groups are over-split, and the dark green cluster shows evidence of “shattering”, in that these cells cannot be re-assigned to the proper clusters even with iterative re-merging of clusters. **b** Schematic of the chooseR approach. After 100 randomly drawn subsampled sets of cells are clustered, a cell–cell co-clustering frequency matrix is used to calculate mean per-cluster silhouette scores. This process is repeated for each parameter value of interest. **c** Under-, optimal, and over-clustering illustrated on a single-cell RNAseq dataset containing ~ 11,000 PBMCs. **d** Silhouette distribution plot for the dataset in c. Each dot represents a cluster at a given clustering parameter value. Medians with 95% CI are shown for each parameter value. The vertical red line marks the optimal resolution (in the Seurat package), and the blue line marks the decision threshold (“Methods” section). **e** Median silhouette scores for each cluster at the suggested optimal resolution = 2. Cell type families are indicated by colored boxes along the x-axis. This identifies which clusters are likely less robust at the optimal parameter value and should be investigated further in a more focused way

In recent years, single-cell RNA sequencing (scRNA-seq) has developed into a powerful tool for defining and characterizing cell types [1–7] by profiling many individual cells at scale and analysing their gene expression to find patterns of variation. This generally requires clustering cells, and many different approaches and software packages have been developed to streamline this process [8–11]. Despite recent advances and adaptations applied to clustering algorithms [12], choosing appropriate clusters (Fig. 1a) remains challenging as: (1) many algorithms have tuneable parameters whose user-defined adjustment can produce very different results; and (2) without foreknowledge of cell types, it is hard to address the quality of the chosen clusters, and whether the cells have been under- or over-clustered (Fig. 1a). In general, under-clustering occurs when clusters are too broad and mask underlying biological structure. Near-optimal clustering is when most clusters relate to known or presumed cell types, with relevant biological distinctions revealed and without noisy, unreliable, or artifactual sub-populations. When cells are slightly over-clustered, non-relevant subdivisions have been introduced; however, these subclusters can still be merged to recover appropriate cell types. Once severe over-clustering occurs, however, some clusters may be shattered, meaning they are segregated based on non-biological variation to the point where iterative re-merging cannot recover the appropriate cell types. Although certain hierarchical approaches, by definition, can avoid this shattering phenomenon, strict hierarchical clustering (in contrast to hierarchical assembly of clustered cell types) is not commonly used in most single-cell RNA-seq analysis workflows.

A number of data-driven metrics have been proposed to address the challenges of over- and under-clustering, some of which estimate the statistical robustness of clusters. A notable example is the *SC3* package, which provides an estimate of cluster number in a dataset and also includes a measure of cluster stability [13]; however, its current implementation is optimized for smaller datasets, and cannot readily be used with other methods outside of the *SC3* package. Alternative approaches include testing clustering across various parameter regimes and creating a merged consensus clustering, or iteratively applying a machine learning classifier to identify clusters as candidates for re-merging [14–17]. However, these approaches are all post hoc, will not split clusters in the case of under-clustering, and assume that over-clustered populations can be hierarchically merged into properly clustered cell types, which is not always the case (Fig. 1a).

To address this gap, we developed chooseR, a framework that uses iterative, sub-sampled clustering across algorithm parameters to guide decisions about optimal clustering parameter values and calculate cluster robustness (Fig. 1b). Examples of common parameters it can optimize include the resolution (in the Seurat and scVI packages), k (for k-means clustering approaches), or the number of reduced dimensions for manifolds on which the cells are projected. This framework is designed to be used during the initial clustering phase with any clustering method on single cell datasets regardless of size, depth, tissue of origin, or species. Crucially, because it wraps around the user’s clustering workflow of choice, it allows for unbiased as well as weighted clustering using known marker genes, if such prior information exists for the system in question and can be incorporated into the clustering workflow being used. In addition, it not only returns the near-optimal set of parameter values, but also identifies which clusters are less robust and require more focused analysis and re-clustering in isolation; this is important  because even a globally optimal parameter set does not necessarily imply that all clusters are equally well-resolved.

## Results

To demonstrate under-, near-optimal, and severe over-clustering of real cellular data, we used a dataset of ~ 11,000 human peripheral blood mononuclear cells (PBMCs) from the 10x Genomics public repository. When this dataset is under-clustered with the Seurat workflow (Fig. 1c, left), sub-types of B cells and dendritic cells are not resolved, and subsets of CD4+ and CD8+ T cells are called as being in the same cluster. By contrast, near-optimal clustering (Fig. 1c, middle) accurately reflects known cell types and subtypes. As the clustering parameter (here, the resolution value) is increased further (Fig. 1c, right), the data is split into many small clusters, some of which are subsets of larger clusters, but some of which are shattered into intermixed groups, which cannot be re-merged in a coherent way to reconstitute the larger cell groups. This shattering of clusters is also evident in how individual cells are distributed into clusters over a range of resolutions, as revealed by the Clustree tool (Additional file 1: Fig. S1). Initially, at low or moderate resolution values, the cell membership of large clusters divides into more refined branches, but with further increases of resolution and cluster number, significant proportions of cells intermix across major cell types.

We applied our chooseR framework to identify de novo a set of parameter values that produces near-optimal clustering of this dataset (Fig. 1d). When used with the Seurat package [8, 9], chooseR identified a resolution of 2 as the value with most clusters (red line), based on confidence-interval bounds on the median silhouette value distributions (blue line) over all parameter values (“Methods” section). At this resolution, the cluster silhouette scores provide an intuitive metric for identifying highly robust clusters and those with lower robustness, the latter of which may require further investigation (Fig. 1e). Overall, however, the resolution value returned by chooseR results in clustering that corresponds well with known cell types (Fig. 1c, middle), demonstrating that this data-driven approach yields reliable clustering parameter values.

It is rare that a single set of parameters in any clustering algorithm will resolve all putative cell types equally well, especially given the multi-scale organization of most biological systems. Thus, we highlight that an important aspect of chooseR is its ability to identify which clusters are candidates for re-merging, followed by re-clustering in isolation in order to better subdivide these cells into more robust groups. As an example of this strategy, we selected poorly-resolved clusters in the analysis as candidates for further investigation, and reclustered them in isolation from other cells to identify better subdivisions among them (Additional file 2: Fig. S2). Finally, there are multiple parameters besides the resolution that affect clustering, and chooseR can be used to select near-optimal values for all of them, with the added benefit of showing which ones appear to have the largest effects on clustering (Additional file 3: Fig. S3).

Whereas the illustrations in Fig. 1 are all based on 100 iterations, each subsampling 80% of the cells, chooseR returns the same near-optimal resolution parameter value at 50 iterations, and using only 50% of the cells at each iteration (Additional file 3: Fig. S3). Although it is not recommended to use too low of a subsampling percentage or too few iterations, the user can reduce these numbers to speed up the overall computational time, which scales linearly with the number of iterations; the scaling with respect to the number of cells depends on the underlying clustering algorithm being used. Regardless of the number of iterations or subsampling percentage, however, chooseR provides an assessment of per-cluster robustness at the resulting value of the near-optimal parameter, which the user can ultimately evaluate.

**Fig. 2:**
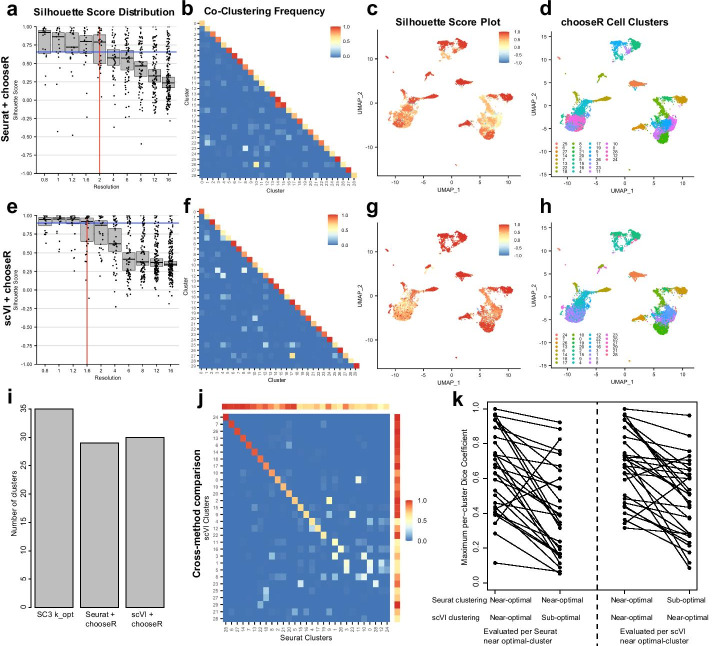
chooseR generalizes across different clustering algorithms. **a**–**d** Results of applying chooseR using Seurat to a data set comprising 11,000 human PBMCs. **a** Silhouette score distribution showing the selection of the optimal clustering parameter (resolution) value. Each dot represents a cluster. Medians with 95% CI are shown for each resolution. The vertical red line marks the optimal resolution, and the horizontal blue line marks the decision threshold (“Methods” section). **b** Average co-clustering frequency at the optimal resolution value = 2, following clustering on 100 random sub-samples of the data using 80% of the cells. **c** UMAP representation of cells, colored by silhouette score at the suggested optimal resolution value = 2. **d** Same as c, but colored by predicted cluster. **e**–**h** Same as **a**–**d**, but using the scVI workflow for  clustering. Here, the optimal resolution value = 1.6. UMAP coordinates in **c**, **d**, **g**, **h** are calculated in  Seurat with 50 principal components. UMAP coordinates from scVI, colored by silhouette scores and clusters are shown in Additional file 4: Fig. S4. **i** Bar plot comparing the number of recommended clusters using chooseR with Seurat and with scVI, as well as the recommended optimal cluster number (k) from the SC3 algorithm. **j** Heatmap showing the Dice coefficients between suggested Seurat and scVI clusters at optimal parameter values, indicating good overlap between the two partitionings. Colorbars on the top and right side of the heatmap indicate the within-cluster co-clustering score for each Seurat or scVI-derived cluster, showing a general trend that clusters with lower maximum Dice coefficient values are also the ones that are difficult to resolve clearly with either method. **k** Maximum Dice coefficients per cluster when comparing optimal versus sub-optimal parameter sets for clustering with Seurat and scVI. The two sets of dots (joined by lines) on the left indicate the maximum Dice coefficients per cluster obtained using Seurat with optimal parameters when compared to scVI-derived clusters using optimal (left) and sub-optimal parameter sets. The sets of dots on the right are the converse, showing the maximum Dice coefficient per scVI-derived cluster. Most clusters are less well-reproduced across Seurat and scVI when one or the other method is run with sub-optimal parameters, indicating that chooseR’s ability to identify near-optimal parameters leads to better reproducibility of clusters across different clustering algorithms

Next, we showed that chooseR generalizes across widely-used clustering workflows by applying it to the same dataset using the Seurat package and the scVI workflow integrated into Scanpy [10, 11]. Whereas Seurat uses PCA for dimensionality reduction, scVI uses deep neural networks to encode the transcriptome in a low-dimensional latent vector space. They both subsequently use different community detection-based approaches (the Louvain and Leiden algorithms, respectively) with tunable parameters to identify a suitable set of clusters. Here, chooseR identified a resolution parameter of 2 as near-optimal for Seurat, yielding 29 clusters, and identified a resolution parameter of 1.6 as near optimal for scVI, yielding 30 clusters (Fig. 2a, e). At their respective parameter values, both workflows identified clusters with a broad range of co-clustering frequencies with some off-diagonal hits, suggestive of populations of mixed robustness (Fig. 2b, f). The silhouette scores show that areas of differing robustness coincide well across the two methods (Fig. 2c, g and Additional file 4: Fig. S4), and the near-optimal clusters from Seurat and scVI are in good agreement (Fig. 2d, h), despite having different near-optimal values for their respective resolution parameters. In addition, the silhouette scores per cluster correspond well to the within cluster co-clustering scores along the diagonal (Additional file 4: Fig. S4), reflecting their shared information content. Separately, the *SC3* package [13], which has a built-in consensus-based approach to find an optimal k-means clustering, recommended an optimal k-value of 35 clusters (Fig. 2i). Thus, the generalized consensus approach in chooseR yields the same clusters using multiple clustering algorithms, highlighting its broad applicability and compatibility with a user’s preferred clustering package (Fig. 2j). In particular, the near-optimal parameters selected by chooseR for the Seurat and scVI workflows yield clusters that agree better with each other (based on per-cluster maximum Dice coefficient metrics) than with the partitions obtained using sub-optimal parameter sets for either method (Fig. 2k). Thus, although no official cell type annotations were included as part of the release of this dataset, the consistency of clusters given by the chooseR-selected optimal parameters in Seurat and scVI supports the notion that these clusters likely reflect robust aspects of the underlying biological diversity.

**Fig. 3:**
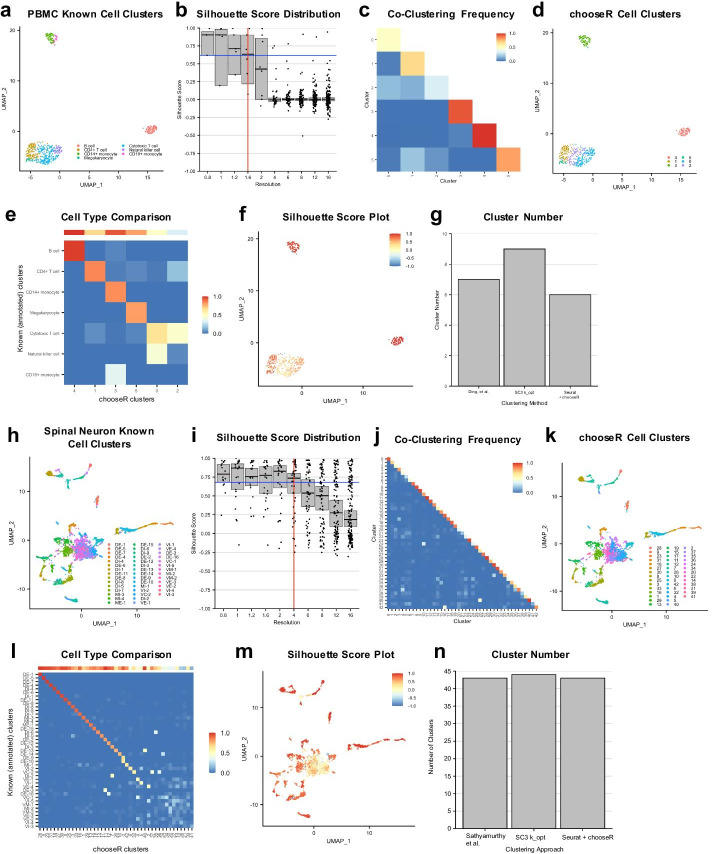
chooseR recovers annotated cell types in datasets of varying size and complexity. **a**–**g** Results of using chooseR  and Seurat on the Ding et al. [18] Smart-Seq human PBMC dataset. **a** UMAP with cells colored by published, annotated clusters. **b** Silhouette distribution plot over multiple resolution parameter values, with each dot representing a cluster. Median with 95% CI is shown for each resolution. The vertical red line marks the optimal resolution, and the horizontal blue line marks the decision threshold (“Methods” section). The optimal parameter value (resolution) = 1.6. **c** Heatmap of average co-clustering frequency per cluster, following clustering on 100 random subsets of the data, each comprising 80% of the total cells. **d** UMAP displaying the optimal cluster labels at the chosen resolution. **e** UMAP displaying the per-cell silhouette scores at the chosen resolution. **f** Heatmap comparing the correlation between clusters at the optimal parameter value and known cell types, colored by the Dice coefficient. The horizontal bar at the top represents the within-cluster co-clustering frequency for each chooseR-derived cluster. **g** Bar plot comparing the number of recommended clusters across methods. **h**–**n** As in **a**–**g** but applied to the Sathyamurthy et al. [7] mouse spinal cord dataset

Next, we tested chooseR on a dataset with fewer cells profiled more deeply, applying it to identify the optimal Seurat resolution parameter on a published Smart-Seq-based PBMC dataset [18]. Seven cell types, including three T-cell subtypes and 2 monocyte subtypes, were annotated in the original dataset, based on cell type clustering and assignment from a much larger dataset (Fig. 3a). chooseR suggested a Seurat resolution value of 1.6 as near-optimal for this dataset (Fig. 3b) and identified clusters with a range of co-clustering frequencies at this resolution (Fig. 3c). The suggested clusters agree well visually with those annotated in the original dataset (Fig. 3d, e), though some mixing is evident (Fig. 3e). Specifically, this resolution value results in a single cluster for all monocytes, rather than the annotated two, and mixes cytotoxic T cells and natural killer cells across two clusters (Fig. 3e). However, the silhouette scores clearly reveal low robustness within the clusters encompassing these mixed cell populations (Fig. 3f), which is further reflected in the co-clustering frequency matrix (Fig. 3c), the dot plot of cluster robustness (Additional file 5: Fig. S5) and silhouette UMAP projection (Fig. 3f). This highlights the ability of chooseR to mark subgroups of cells and clusters that require further investigation. It is worth noting that the published cluster annotations were generated on a substantially larger dataset that was not included here [18], so the overall concordance between clusters and cell types (Fig. 3e), and cluster number (Fig. 3g), demonstrates the efficacy of chooseR in identifying biologically meaningful clusters, even when applied in isolation on datasets with smaller numbers of cells.

We next applied chooseR to data from tissue with high cellular diversity and more complex relationships between cell types. This mouse adult spinal cord neuron dataset has previously been shown to include distinct dorsal cell types as well as more inter-related ventral cell types [7, 19] (Fig. 3h). Here, chooseR suggested a Seurat resolution value of 4 (Fig. 3i) and identified clusters with widely ranging co-clustering frequencies, suggestive of a mixture of highly distinct and less distinct cell types (Fig. 3j). Visually, the suggested clusters at this resolution agreed well with the 43 types annotated in the original dataset (Fig. 3k). Overall, the recommended clusters strongly correlated with the published clusters, particularly among the more distinct dorsal excitatory and dorsal inhibitory neurons (Fig. 3l). The silhouette score map clearly reveals robust and less robust clusters, the latter concentrated in areas corresponding to closely related ventral neuron groups [7, 19] (Fig. 3m, Additional file 6: Fig. S6). In addition, the correlation between chooseR clusters and annotated subgroups in the mid/ventral groups was weaker (Fig. 3l), as expected. Overall, this highlights chooseR’s ability to identify parameter values resulting in biologically meaningful groupings among cell types with varying degrees of discreteness. Finally, the observation that the number of annotated clusters, the recommended optimal k value from *SC3*, and the number of clusters suggested by chooseR coincide almost exactly gives further support to the idea that these clusters capture relevant axes of biological diversity (Fig. 3n).

## Discussion

Here, we have presented chooseR, a subsampling-based framework to help end-users select clustering parameter values a priori. We demonstrate that chooseR can be used with multiple clustering packages and recovers previously known cell type clusters in a high-cell, lower-depth PBMC dataset, a low-cell, higher-depth PBMC dataset, and a larger, more complex dataset of mouse spinal cord neurons. The underlying assumption is that clustering workflow parameter values most likely to uncover the underlying biological diversity are those that generate a large number of clusters with high robustness, though we note that this may result in a potential bias towards minor over-clustering. However, since chooseR also provides simple, easy-to-interpret metrics—the cluster average silhouette score and co-clustering maps—it also identifies those clusters that are likely over-clustered, highlighting them for merging or further analysis. Finally, to maximize the utility of chooseR, it is designed to be wrapped around any existing clustering algorithm to help guide the selection of tuneable clustering parameter values.

## Conclusion

The chooseR tool assists users in selecting clustering parameters for single cell sequencing analysis. By providing a workflow-independent framework to identify a near-optimal initial set of clusters, we allow end-users to choose with confidence whichever analysis tool they prefer, thus generalizing their strategy towards finding biologically meaningful partitionings of single-cell data.

## Methods

### Overview

The goal of this work was to develop a method for automated selection of clustering parameter values that could be applied to a wide variety of clustering workflows and datasets. The chooseR framework begins by identifying partitionings of the full dataset for the lower bound of the clustering parameter values under investigation. Next, it clusters 100 random subsets comprising 80% of the cells. This subsampled clustering generates co-clustering frequency values for every cell pair, which are then used to calculate each cell’s silhouette score on the full (non-subsampled) clustering. To calculate the silhouette score, the distance metric between any two cells is set at 1 minus their co-clustering frequency. Subsequently, per-cell silhouette scores are aggregated for each cluster to generate a per-cluster measure of robustness, and this entire process is then repeated over the full range of clustering parameter values. For each parameter value, the 95% confidence interval (CI) of the distribution of cluster silhouette scores is calculated. The near-optimal clustering parameter value is the one yielding the highest number of clusters whose median silhouette score overlaps with the 95%CI with the highest lower bound (see details below). This approach has two key outputs: it identifies a near-optimal partitioning for cell type identification and provides a robustness score for each cluster in that partitioning, which can be used to identify low-robustness clusters for further analysis.

### Data sets

We used three publicly available data sets for the analysis presented here:

1. The 10 k PBMCs from a Healthy Donor dataset is publicly available from 10 × Genomics® and may be downloaded at: http://cf.10xgenomics.com/samples/cell-exp/3.0.0/pbmc_10k_v3/pbmc_10k_v3_filtered_feature_bc_matrix.tar.gz.
  After making variable names unique, any gene not expressed in at least 3 cells was dropped. Additionally, cells with > 20% mitochondrial reads, < 200 genes detected, or > 5200 genes detected were removed.
2. The Smart-Seq PBMC dataset was published in Ding et al. [18]. It is publicly available through the Broad Institute’s Single Cell Portal (Study Number SCP424). No additional QC metrics or filtering were run, as only those cells with cluster labels in the original study were retained.
3. The mouse spinal cord neuron dataset was published in Sathyamurthy et al. [7]. It is publicly available through the NCBI Gene Expression Omnibus (Ascension Number GSE103892). No further QC metrics or filtering were run, as only those cells with cluster labels in the original study were retained.

### Clustering algorithms

The chooseR package serves as a wrapper that is suited to any individual clustering method. Here, we illustrate the general workflow used with two popular packages for single-cell clustering analyses:

1. Seurat—when using the Seurat package (version 3.1.4), before clustering, the *Seurat::SCTransform* function was used with default parameters to normalize and scale the data, as well as regress out the percentage of mitochondrial genes. Then, *Seurat::RunPCA* was called on the “SCT” assay with 100 PCs, and all other parameters set at default values. Clustering with the Seurat package was performed on all calculated principal components, using the *Seurat::FindNeighbors* function with default parameters, followed by the *Seurat::FindClusters* function with default parameters at the specified resolution.
2. scVI—when using scVI (version 0.6.5) with Scanpy (version 1.4.6), before clustering, a copy of the raw data was saved. Next, the *scanpy.pp.normalize_total* and *scanpy.pp.log1p* functions were used to normalize and scale the data. Then, the 3,000 most highly variable genes were determined using *scanpy.pp.highly_variable_genes* using the Seurat settings, with all parameters at default. Next, the raw data matrix was subset to contain only highly variable genes, before calculating 10 latent vectors for 400 epochs with a helper function provided by scVI. Clustering with the Scanpy package was performed on the latent variables calculated by scVI, using the *scanpy.pp.neighbors* function with default parameters, followed by the *scanpy.tl.leiden* function with default parameters at the specified resolution.

### Workflow

For each of these two packages, the general chooseR workflow begins with a full clustering performed (as described above) on all cells to establish reference labels for a given resolution. This is followed by 100 repetitions of the clustering, each performed on a random 80% subset of the dataset, drawn without replacement. From the 100 subsampled clustering runs, a pairwise cell–cell co-clustering matrix is created where each entry indicates the co-clustering frequency between each pair of cells. The co-clustering frequency *f*(*x,y*) between two cells, x and y, is defined as the number of runs in which the cells were grouped into the same cluster divided by the number of times the cells were both included in a subsampling run. This co-clustering matrix is converted to a distance matrix by subtracting all values from 1; this leads to a distance value of 0 for cell pairs that always co-clustered and a value of 1 for cell pairs that were never grouped into the same cluster. Using this distance matrix, silhouette scores are then calculated per cell to each cluster. These per-cell silhouette scores are calculated and averaged across the full clustering labels to produce a mean score per cluster. The distribution of these per-cluster silhouette scores represents the robustness of the partitioning at a given parameter value. This entire process is then repeated over a range of clustering parameter values.

To determine the near-optimal value of the clustering parameter(s), 95% confidence intervals were calculated on the median silhouette score for each clustering parameter value using the CRAN package *boot* with 25,000 re-samples and “type = ‘bca’”. The decision threshold was defined as the highest value of all lower ends of the 95% confidence intervals. The near-optimal clustering parameter value was then selected as the one yielding the largest number of clusters, while still having a median per-cluster silhouette score above the decision threshold. Finally, for comparison with the optimal k value obtained from the SC3 package, we used the *SC3::sc3_estimate_k* function with default parameter values.

## Supplementary information

**Additional file 1: Fig. S1.** Clustree displaying the problem of under-, near-optimal, and over-clustering. Generated with Clustree v0.4.2 from CRAN. As resolution increases, the number of crossing arrows increases, indicative of shattering—that is, clustering on noise that could not be solved by hierarchical merging.

**Additional file 2: Fig. S2.** Illustration of subclustering framework for better resolution of poorly-resolved clusters. For clusters identified as being poorly-resolved in the top-level clustering using all cells (left panel), it is possible to rerun the chooseR framework with Seurat on just these cells, following the same general procedure. This improves the clustering of certain subsets of cells (arrowheads in the lower right panel), allowing for them to be better resolved than they were at the top-level clustering. Although there is no guarantee that all cells will eventually be sorted into the clusters with the same robustness (due to biological and technical noise in the data set), successive reclustering of poorly resolved clusters at the top level can help to refine some cell clusters.

**Additional file 3: Fig. S3.** Application of chooseR to additional clustering parameters and effect of repetitions and subsampling on chooseR results. **a** Silhouette distribution plot from chooseR applied to the Seurat parameter determining how many variable genes should be selected for clustering cells (on the PBMC data set explored in Fig. 2). The red line reflects the near-optimal value identified by chooseR, and the blue line reflects the decision threshold. Seurat returns relatively robust clusters somewhat independently of this parameter. **b** Cluster silhouette scores for the near-optimal value of the number of highly variable genes selected for clustering. **c** Same as **a**, but for the Seurat parameter determining how many principal components to use for clustering. Here, the cluster robustness stabilizes after selecting more than 20 PCs for this data set. **d** Same as **b**, but for the near-optimal value of the number of PCs used for clustering. **e** Silhouette distribution plot over different values of the resolution parameter when using only 20% of the total cells in each iteration. The near-optimal parameter value for the resolution is 4, which differs from that identified when subsampling 80% of all cells in each iteration (Fig. 2). **f** Silhouette scores for each cluster at the near-optimal resolution value identified in **e**. The overall scores are substantially lower than when using 80% of all cells in each iteration (Fig. 2), suggesting that subsampling only 20% of all cells per iteration is too few. **g**, **h** Same as **e–f**, but subsampling 50% of all cells per iteration. Here, the near-optimal value of the resolution parameter is the same as when subsampling 80% of all cells, and the silhouette distribution at this parameter value resembles that when using 80% of all cells. **i–l**, Same as **e–h**, but with 20 and 50 iterations using 80% of all cells at each iteration. Here, the near-optimal value of the resolution identified is the same as the one found with 100 iterations (Fig. 2). The time cost for running chooseR scales linearly with the number of iterations, and approximately O(number of cells^1.5^) per iteration using Seurat; this latter scaling is dependent on the underlying clustering approach that is wrapped into chooseR.

**Additional file 4: Fig. S4.** UMAP representations and silhouette scores corresponding to Fig. 2. **a**, **b**, UMAPs calculated from scVI’s latent variables, displaying silhouette score and suggested clusters at resolution equals 1.6. **c** Dot plot displaying individual cluster silhouette scores for chooseR  and Seurat at resolution equals 2. Those with low scores would be candidates for further investigation. **d** As in **c** but with chooseR and scVI at resolution = 1.6. **e**, **f** co-clustering matrices (as in Fig. 2b, f), but reordered to match panels **c** and **d**, showing that clusters with good silhouette scores also have high self co-clustering values, as shown along the diagonal.

**Additional file 5: Fig. S5.** Silhouette scores for Ding et al. [18]. Dot plot displaying individual cluster silhouette scores for chooseR  and Seurat at resolution parameter value = 1.6. Clusters with low scores would be candidates for further investigation.

**Additional file 6: Fig. S6.** Silhouette scores for Sathyamurthy et al. [7]. Dot plot displaying individual cluster silhouette scores for chooseR and Seurat at resolution parameter value = 4. Clusters with low scores would be candidates for further investigation.

## Data Availability

The implementation of this pipeline wrapped onto the Seurat R package, including downstream parameter outputs and visualizations, is freely available on github at: https://github.com/rbpatt2019/chooseR. All data sets used for the analyses here are freely available, with URLs and accession numbers included in the text.

## Abbreviations

scRNA-seq: Single cell RNA sequencing
PBMC: Peripheral blood mononuclear cell
PCA: Principal component analysis
UMAP: Uniform manifold approximation and projection
