# Supplementary figures and images for: Selecting single cell clustering parameter values using subsampling-based robustness metrics
Source: BMC Bioinformatics. 2021 Feb 1;22:39. doi: 10.1186/s12859-021-03957-4 (PMC7852188; doi:10.1186/s12859-021-03957-4)

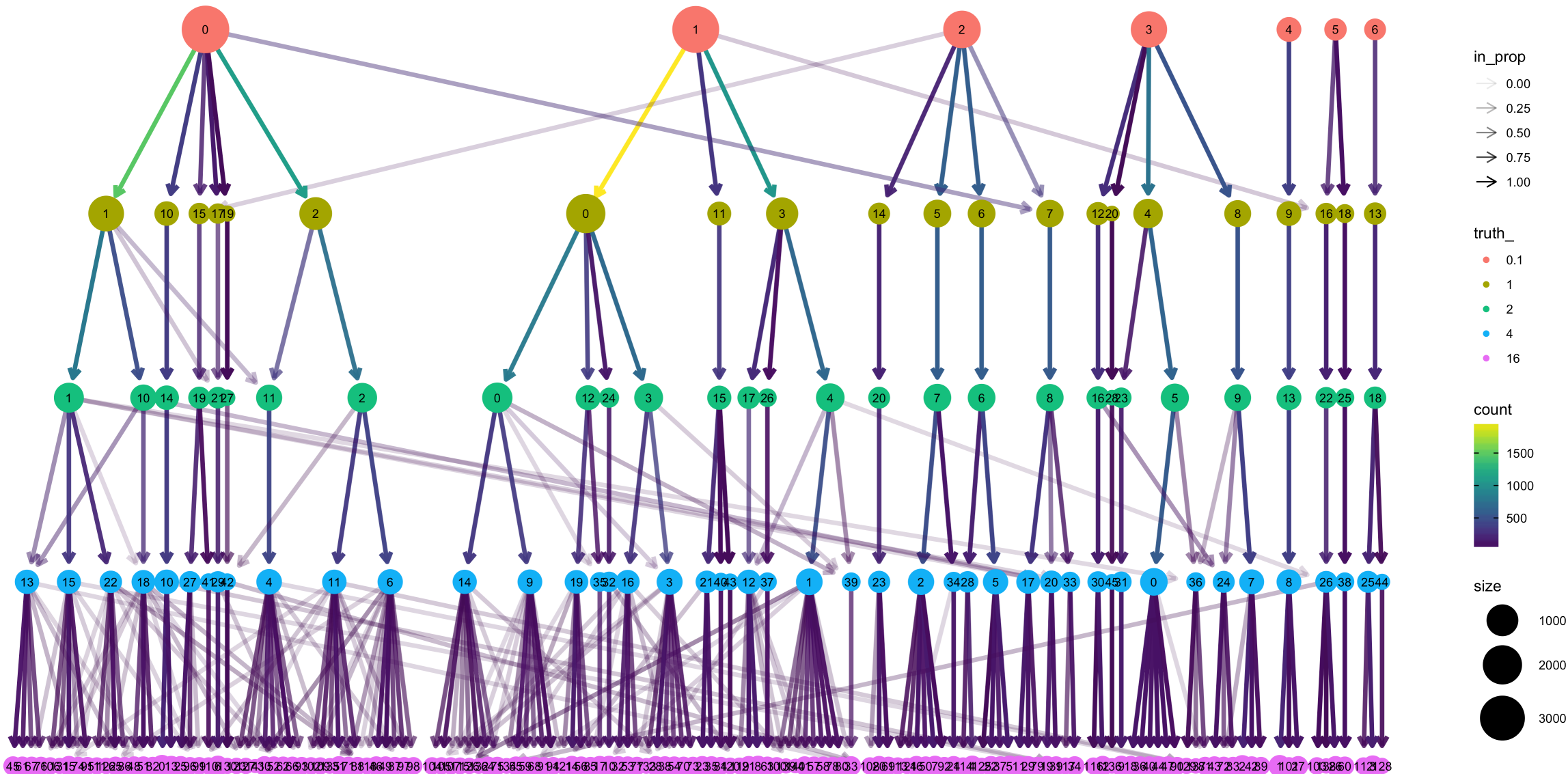

Supplement: Supplementary file 1 — Additional file 1: Fig. S1. Clustree displaying the problem of under-, near-optimal, and over-clustering. Generated with Clustree v0.4.2 from CRAN. As resolution increases, the number of crossing arrows increases, indicative of shattering—that is, clustering on noise that could not be solved by hierarchical merging. [file 12859_2021_3957_MOESM1_ESM.pdf]

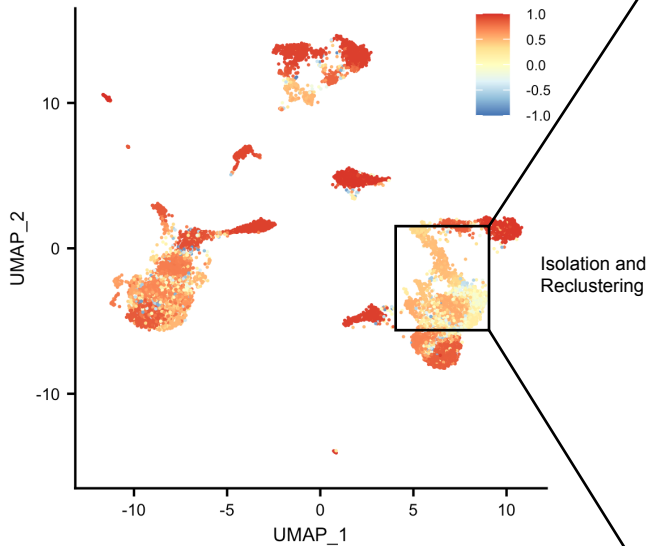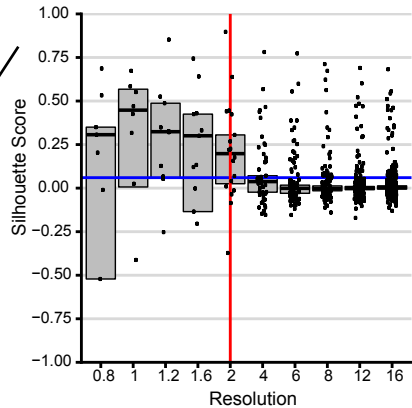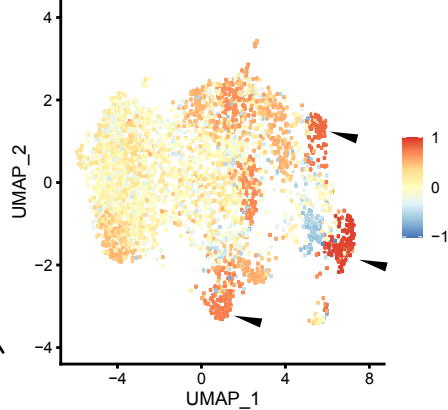

Supplement: Supplementary file 2 — Additional file 2: Fig. S2. Illustration of subclustering framework for better resolution of poorly-resolved clusters. For clusters identified as being poorly-resolved in the top-level clustering using all cells (left panel), it is possible to rerun the chooseR framework with Seurat on just these cells, following the same general procedure. This improves the clustering of certain subsets of cells (arrowheads in the lower right panel), allowing for them to be better resolved than they were at the top-level clustering. Although there is no guarantee that all cells will eventually be sorted into the clusters with the same robustness (due to biological and technical noise in the data set), successive reclustering of poorly resolved clusters at the top level can help to refine some cell clusters. [file 12859_2021_3957_MOESM2_ESM.pdf]

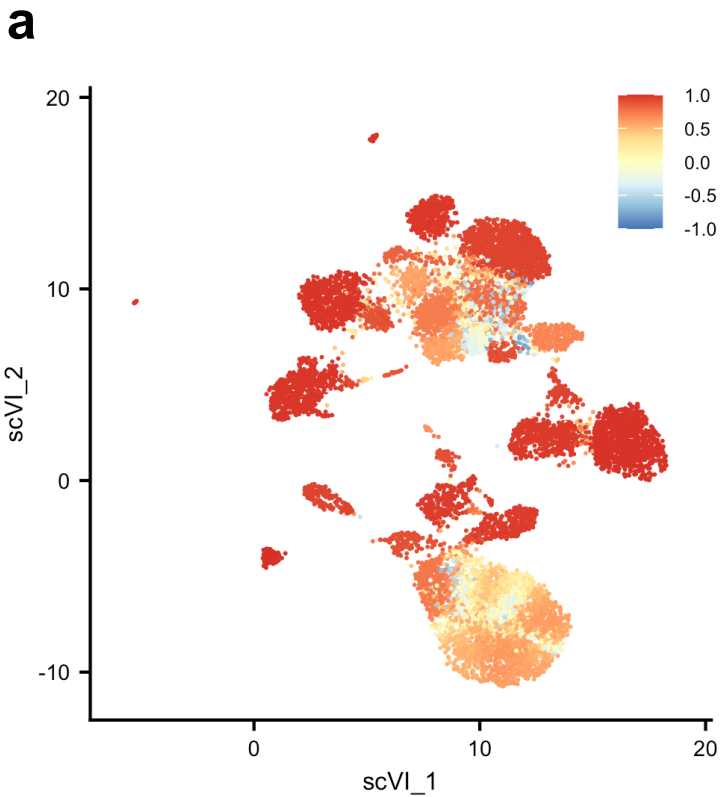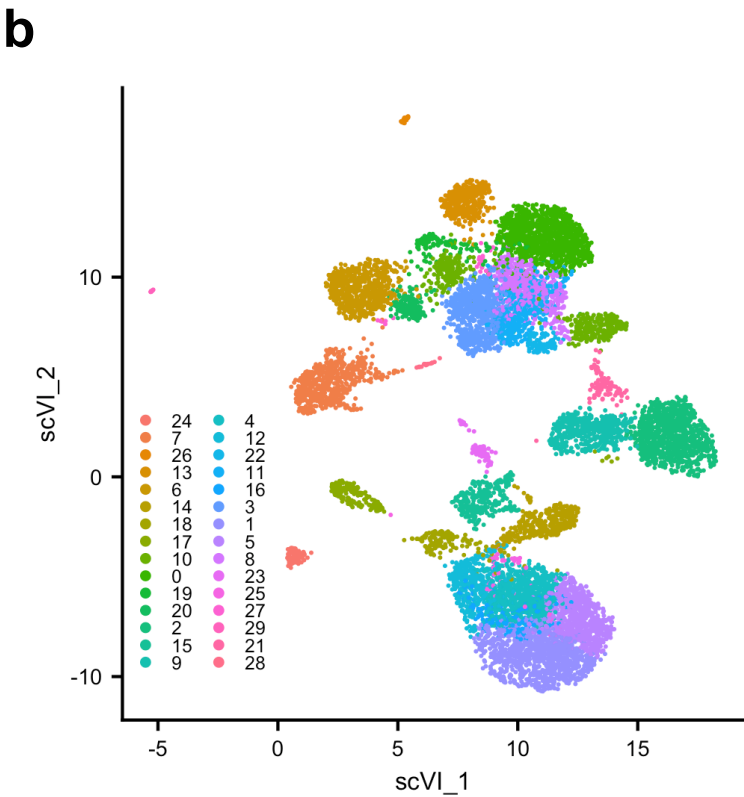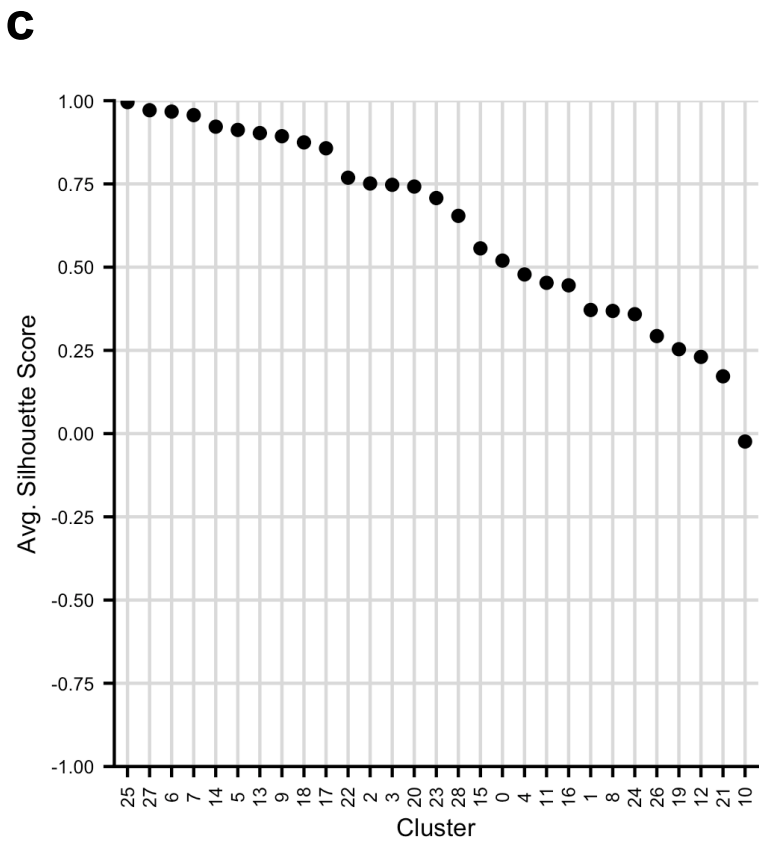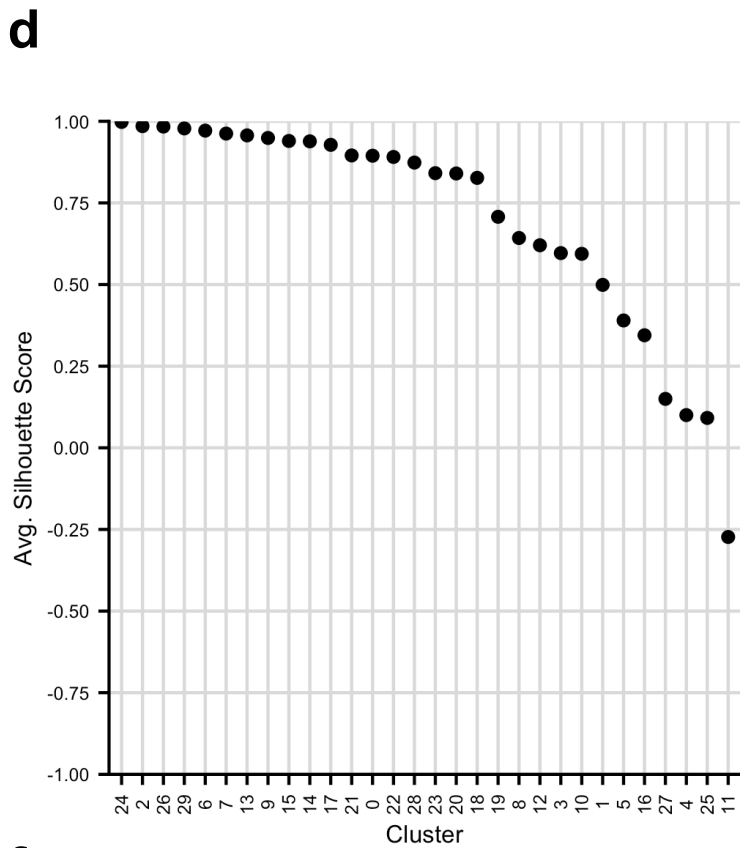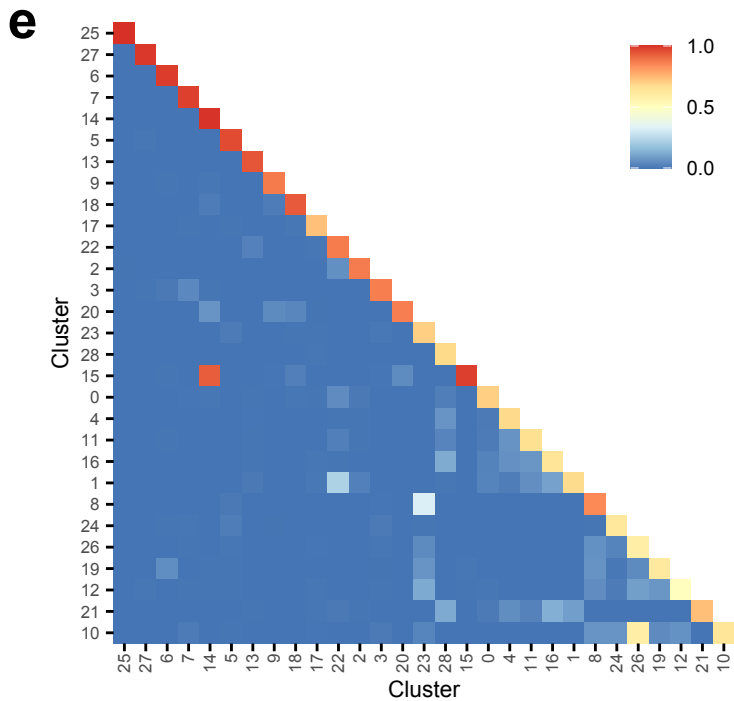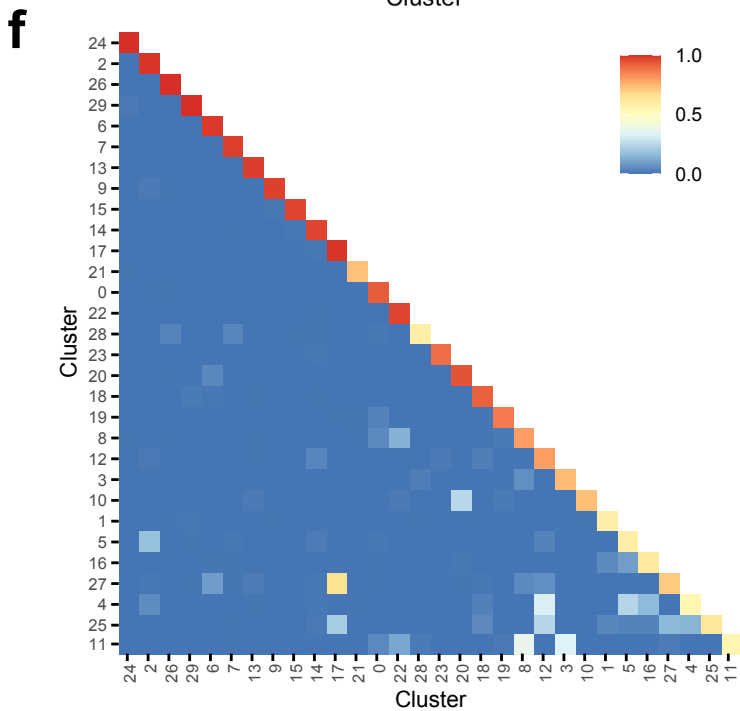

Supplement: Supplementary file 4 — Additional file 4: Fig. S4. UMAP representations and silhouette scores corresponding to Fig. 2. a, b, UMAPs calculated from scVI’s latent variables, displaying silhouette score and suggested clusters at resolution equals 1.6. c Dot plot displaying individual cluster silhouette scores for chooseR and Seurat at resolution equals 2. Those with low scores would be candidates for further investigation. d As in c but with chooseR and scVI at resolution = 1.6. e, f co-clustering matrices (as in Fig. 2b, f), but reordered to match panels c and d, showing that clusters with good silhouette scores also have high self co-clustering values, as shown along the diagonal. [file 12859_2021_3957_MOESM4_ESM.pdf]

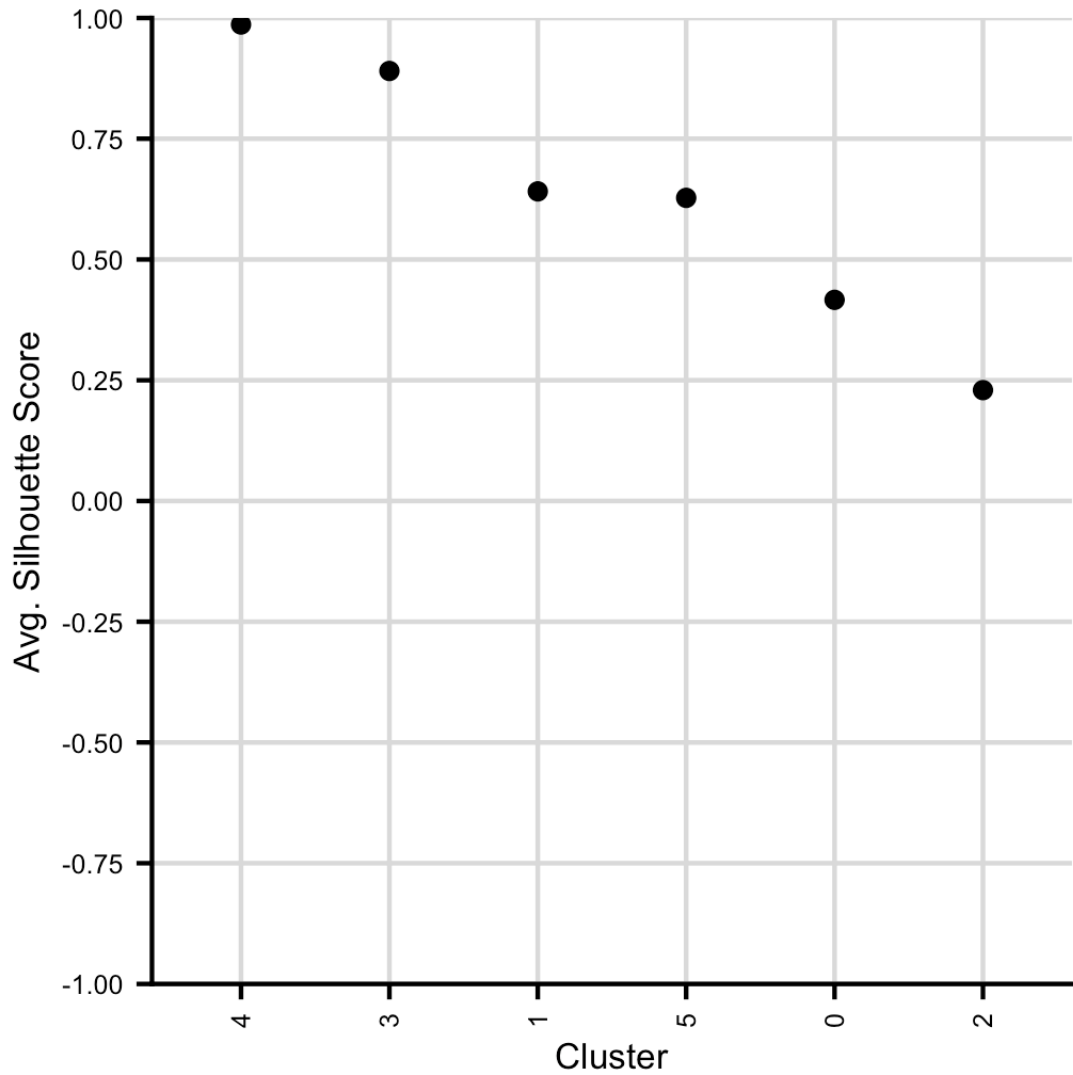

Supplement: Supplementary file 5 — Additional file 5: Fig. S5. Silhouette scores for Ding et al. [18]. Dot plot displaying individual cluster silhouette scores for chooseR and Seurat at resolution parameter value = 1.6. Clusters with low scores would be candidates for further investigation. [file 12859_2021_3957_MOESM5_ESM.pdf]

Avg. Silhouette Score

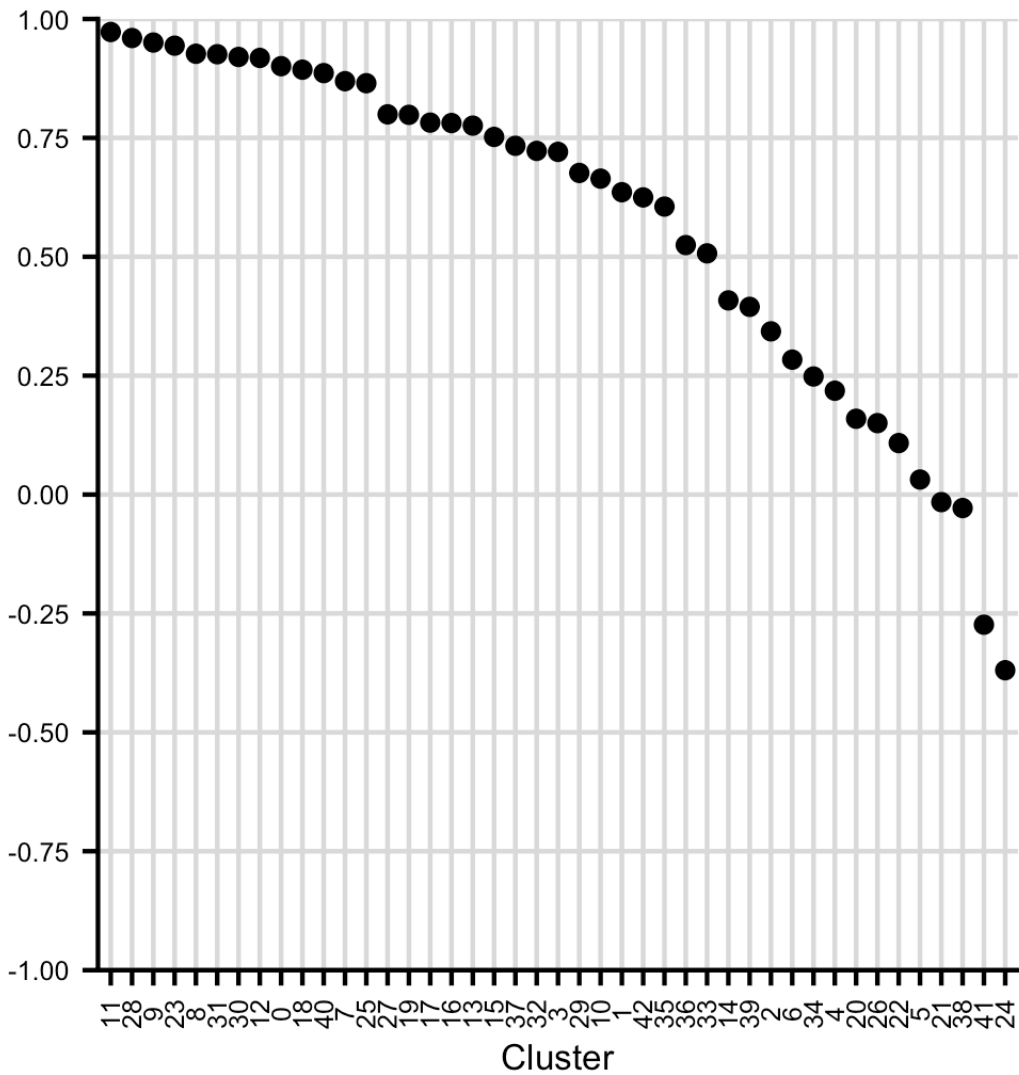

Supplement: Supplementary file 6 — Additional file 6: Fig. S6. Silhouette scores for Sathyamurthy et al. [7]. Dot plot displaying individual cluster silhouette scores for chooseR and Seurat at resolution parameter value = 4. Clusters with low scores would be candidates for further investigation. [file 12859_2021_3957_MOESM6_ESM.pdf]
