# Supplementary material for: Selecting single cell clustering parameter values using subsampling-based robustness metrics
Source: BMC Bioinformatics. 2021 Feb 1;22:39. doi: 10.1186/s12859-021-03957-4 (PMC7852188; doi:10.1186/s12859-021-03957-4)
Supplement: Supplementary file 3 — Additional file 3: Fig. S3. Application of chooseR to additional clustering parameters and effect of repetitions and subsampling on chooseR results. a Silhouette distribution plot from chooseR applied to the Seurat parameter determining how many variable genes should be selected for clustering cells (on the PBMC data set explored in Fig. 2). The red line reflects the near-optimal value identified by chooseR, and the blue line reflects the decision threshold. Seurat returns relatively robust clusters somewhat independently of this parameter. b Cluster silhouette scores for the near-optimal value of the number of highly variable genes selected for clustering. c Same as a, but for the Seurat parameter determining how many principal components to use for clustering. Here, the cluster robustness stabilizes after selecting more than 20 PCs for this data set. d Same as b, but for the near-optimal value of the number of PCs used for clustering. e Silhouette distribution plot over different values of the resolution parameter when using only 20% of the total cells in each iteration. The near-optimal parameter value for the resolution is 4, which differs from that identified when subsampling 80% of all cells in each iteration (Fig. 2). f Silhouette scores for each cluster at the near-optimal resolution value identified in e. The overall scores are substantially lower than when using 80% of all cells in each iteration (Fig. 2), suggesting that subsampling only 20% of all cells per iteration is too few. g, h Same as e–f, but subsampling 50% of all cells per iteration. Here, the near-optimal value of the resolution parameter is the same as when subsampling 80% of all cells, and the silhouette distribution at this parameter value resembles that when using 80% of all cells. i–l, Same as e–h, but with 20 and 50 iterations using 80% of all cells at each iteration. Here, the near-optimal value of the resolution identified is the same as the one found with 100 iterations (Fig. 2). The [file 12859_2021_3957_MOESM3_ESM.pdf]

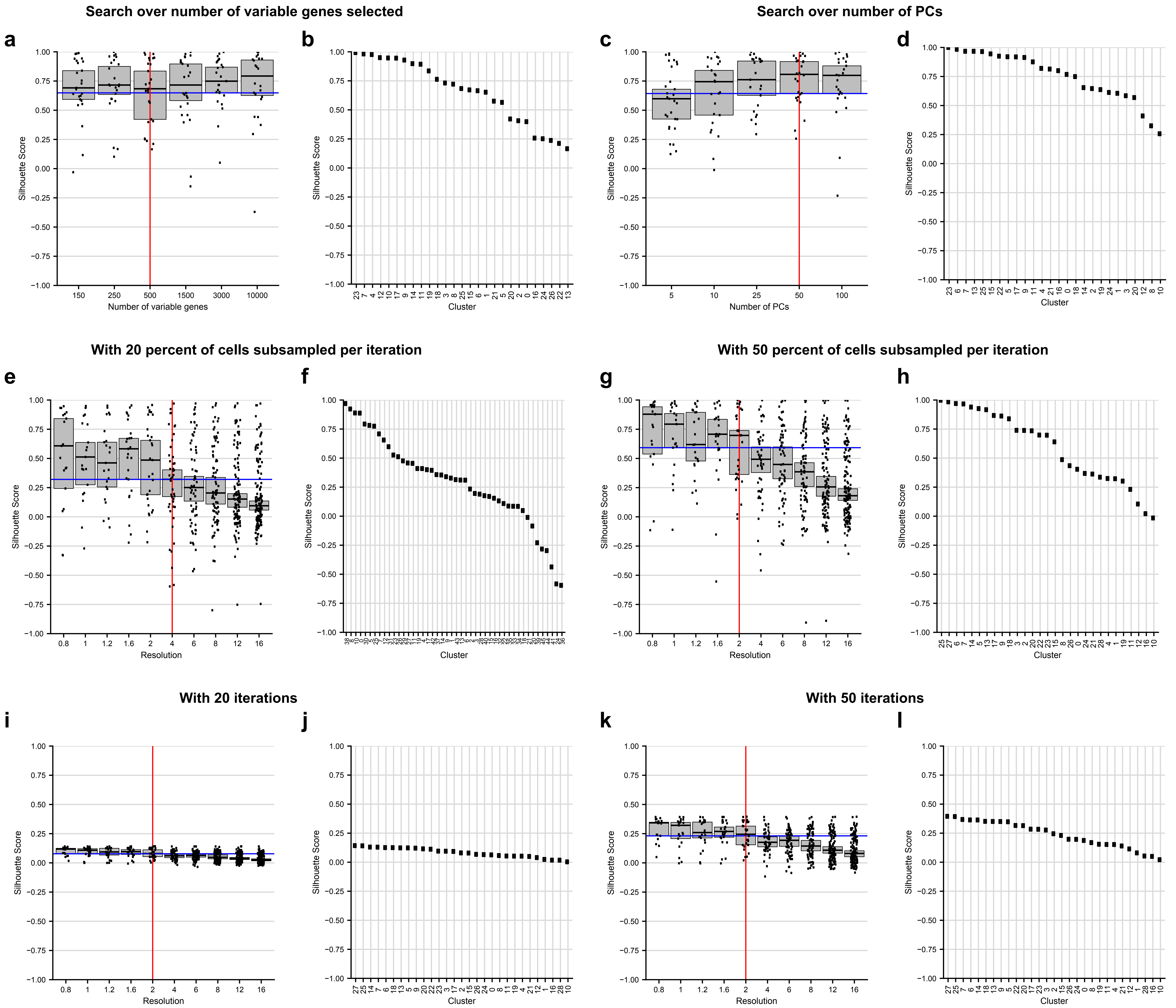

Search over number of variable genes selected
Search over number of PCs
a
b
c
d
Silhouette Score
1.00
0.75
0.50
0.25
0.00
−0.25
−0.50
−0.75
−1.00
150
250
500
1500
3000
10000
Number of variable genes
Cluster
5
10
25
50
100
Number of PCs
With 20 percent of cells subsampled per iteration
With 50 percent of cells subsampled per iteration
e
f
g
h
0.8
1
1.2
1.6
2
4
6
8
12
16
Resolution
With 20 iterations
With 50 iterations
i
j
k
l
